# Therapeutic Plasma Exchange in Critically Ill Children with Neuroimmunological Disorders: A Single-Center Cohort Study

**DOI:** 10.3390/children13070908

**Published:** 2026-07-09

**Authors:** Ebru G. Sahin, Kubra B. Guvenc, Gulcan A. Yucel, Elif Yasar, Fatih Varol, Cansu Durak

**Affiliations:** 1 Department of Pediatric Intensive Care, Sancaktepe Sehit Prof. Dr. Ilhan Varank Training and Research Hospital, University of Health Sciences, Istanbul 34785, Türkiye; 2 Department of Pediatric Neurology, Sancaktepe Sehit Prof. Dr. Ilhan Varank Training and Research Hospital, University of Health Sciences, Istanbul 34785, Türkiye; 3 Department of Pediatric Radiology, Sancaktepe Sehit Prof. Dr. Ilhan Varank Training and Research Hospital, University of Health Sciences, Istanbul 34785, Türkiye

**Keywords:** therapeutic plasma exchange, pediatric neuroimmunology, autoimmune encephalitis, Guillain–Barré syndrome, acute necrotizing encephalopathy, infection-triggered encephalopathy syndrome, pediatric critical care

## Abstract

**Highlights:**

**What are the main findings?**

- Therapeutic plasma exchange (TPE) was used across a broad spectrum of pediatric neuroimmunological disorders in a tertiary pediatric intensive care unit, including conditions beyond current ASFA guideline recommendations, reflecting real-world clinical practice.
- Favorable neurological outcomes were observed in 63.6% of patients, with mortality confined to severe encephalopathic syndromes and no major TPE-related complications.

**What are the implications of the main findings?**

- TPE may represent a feasible and safe escalation therapy for critically ill children with severe or refractory neuroimmunological disorders, even when high-level evidence is lacking.
- Multicenter prospective studies are needed to better define optimal patient selection, treatment timing, and the role of TPE in pediatric neuroimmunology.

**Abstract:**

**Background:** Therapeutic plasma exchange (TPE) is increasingly used in pediatric neuroimmunological disorders; however, evidence remains limited, particularly for rare neuroinflammatory and infection-triggered encephalopathy syndromes. We aimed to describe the clinical characteristics, treatment patterns, and outcomes of critically ill children who underwent TPE for neuroimmunological disorders in a tertiary pediatric intensive care unit. **Methods:** This retrospective observational study included consecutive patients younger than 18 years who received TPE for neuroimmunological disorders between September 2020 and December 2025. Demographic characteristics, disease spectrum, TPE indications, treatment details, neuroimaging findings, cerebrospinal fluid (CSF) characteristics, and clinical outcomes were reviewed. Neurological outcome at hospital discharge was assessed using the Pediatric Cerebral Performance Category (PCPC) score. **Results:** Twenty-two patients were included. The median age was 104 months (IQR, 62.3–151.8), and 50% were female. Guillain–Barré syndrome (GBS) was the most common diagnosis (n = 6), followed by autoimmune encephalitis (n = 6), infection-triggered encephalopathy syndrome (ITES)/acute necrotizing encephalopathy (ANE) spectrum disorders (n = 4), myelin oligodendrocyte glycoprotein antibody-associated disease (MOGAD) (n = 3), acute disseminated encephalomyelitis (ADEM) (n = 2), and mild encephalitis/encephalopathy with a reversible splenial lesion (MERS) (n = 1). The median interval from symptom onset to TPE initiation was 9 days (IQR, 6–13), and patients underwent a median of 7 TPE sessions (IQR, 5–10.5). Nine patients (40.9%) required invasive mechanical ventilation and four (18.1%) required vasoactive support. Favorable neurological outcomes at hospital discharge were observed in 63.6% of patients, whereas 36.4% had unfavorable neurological outcomes. Mortality occurred in 13.6% of patients and was confined to severe encephalopathic syndromes. No major TPE-related complications were observed. **Conclusions:** TPE was used across a heterogeneous spectrum of pediatric neuroimmunological disorders, including both guideline-supported and non-standard indications. Approximately one-quarter of patients had conditions not specifically addressed in current ASFA recommendations, highlighting the gap between real-world clinical practice and the available evidence base. Multicenter studies are needed to better define the optimal role, timing, and patient selection criteria for TPE in pediatric neuroimmunology.

## 1. Introduction

Neuroimmunological disorders represent a diverse group of inflammatory diseases affecting the central and peripheral nervous systems. In children, these conditions include autoimmune encephalitis (AE), acute disseminated encephalomyelitis (ADEM), myelin oligodendrocyte glycoprotein antibody-associated disease (MOGAD), Guillain–Barré syndrome (GBS), and a spectrum of infection-triggered encephalopathy syndromes. Although these disorders differ in pathophysiology and clinical presentation, many share immune-mediated mechanisms that may result in severe neurological dysfunction, prolonged intensive care requirements, and significant long-term morbidity [1,2].

Current treatment strategies are primarily based on immunomodulation. High-dose corticosteroids, intravenous immunoglobulin (IVIG), and disease-specific immunosuppressive agents constitute the mainstay of therapy for most pediatric neuroinflammatory disorders [3,4]. However, a subset of patients experience rapidly progressive disease, refractory neurological deterioration, or life-threatening complications despite first-line treatment. In these situations, therapeutic plasma exchange (TPE) is increasingly considered as a rescue or escalation therapy [5].

TPE exerts its therapeutic effect through the removal of circulating autoantibodies, immune complexes, complement components, cytokines, and other inflammatory mediators. Consequently, TPE has become an established treatment option for several immune-mediated neurological disorders. According to the most recent American Society for Apheresis (ASFA) guidelines, TPE is recommended as a first-line therapeutic modality for selected neurological conditions, particularly Guillain–Barré syndrome, while evidence supporting its use in other pediatric neuroimmunological diseases remains more limited [6]. For many rare neuroinflammatory disorders, especially infection-triggered encephalopathy syndromes, treatment decisions continue to rely largely on pathophysiological rationale, institutional experience, and expert opinion.

Despite the increasing use of TPE in pediatric neurology, the available evidence supporting its use in pediatric neuroimmunological disorders remains limited, and real-world data across a heterogeneous spectrum of diseases are scarce [5,7]. Existing reports have predominantly focused on individual diagnostic categories, whereas critically ill children requiring intensive care frequently represent a heterogeneous population with overlapping neuroimmunological and neuroinflammatory conditions. Consequently, data regarding the utilization, timing, and outcomes of TPE across the broader spectrum of pediatric neuroimmunological disorders remain limited.

The aim of this study was to describe the clinical characteristics, indications, treatment patterns, and outcomes of children who underwent TPE for neuroimmunological disorders in a tertiary pediatric intensive care unit. In addition, we sought to characterize real-world TPE utilization across a heterogeneous spectrum of pediatric neuroimmunological diseases, including both guideline-supported and non-standard indications. By examining disorders within and beyond current ASFA recommendations, we aimed to provide insight into how TPE is applied in contemporary pediatric neurocritical care when evidence is limited and treatment decisions frequently rely on multidisciplinary expert judgment.

## 2. Methods

### 2.1. Study Design and Setting

This retrospective observational study was conducted in the PICU of Sancaktepe Sehit Prof. Dr. Ilhan Varank Training and Research Hospital, a tertiary referral center in Istanbul, Türkiye. All pediatric patients who underwent TPE for neuroimmunological disorders between September 2020 and December 2025 were screened for eligibility.

The study protocol was approved by the local ethics committee (approval number: E-46059653-050.04-308318809; date: 16 March 2026). Due to the retrospective design, the requirement for informed consent was waived.

### 2.2. Patient Selection

Consecutive patients younger than 18 years of age who received TPE for a neuroimmunological disorder during PICU admission between September 2020 and December 2025 were included.

Diagnoses were established by experienced pediatric neurologists and confirmed through multidisciplinary review. Diagnostic classification was based on established international consensus criteria and published guideline recommendations relevant to each disease entity, incorporating clinical, radiological, laboratory, electrophysiological, and antibody findings when applicable. Autoimmune encephalitis cases fulfilled the International Encephalitis Consortium and pediatric autoimmune encephalitis criteria whenever sufficient data were available. Infection-triggered encephalopathy syndromes were classified according to recently published international consensus definitions. ITES phenotypes were further subclassified as acute necrotizing encephalopathy (ANE), acute encephalopathy with biphasic seizures and late reduced diffusion (AESD)-like encephalopathy, mild encephalitis/encephalopathy with a reversible splenial lesion (MERS), or other infection-triggered encephalopathy syndromes, according to the international consensus framework proposed by Sakuma et al. [1,2].

The study population included patients with AE, ADEM, MOGAD, GBS, ANE, infection-triggered encephalopathy syndrome (ITES), MERS, and related neuroinflammatory disorders.

Patients who underwent TPE for non-neurological indications were excluded.

### 2.3. Data Collection

Electronic medical records were reviewed retrospectively. The following variables were collected: demographic characteristics (age and sex), duration of symptoms before admission, admission Glasgow Coma Scale (GCS) score, Pediatric Risk of Mortality III (PRISM III) score, Pediatric Logistic Organ Dysfunction-2 (PELOD-2) score, requirement for invasive mechanical ventilation (IMV), requirement for vasoactive support, neuroimaging findings, cerebrospinal fluid (CSF) characteristics, neuronal antibody results, timing of TPE initiation, number of TPE sessions, PICU length of stay, neurological outcome at discharge, and mortality [8,9]. PRISM-III was used because it was the severity scoring system routinely implemented in our PICU throughout the study period and was automatically recorded within the hospital Health Information System (HIS). Given the retrospective design, PRISM-IV variables were not consistently available for all patients, precluding retrospective calculation of PRISM-IV scores. Serum and/or CSF neuronal antibody testing was performed in patients with suspected central nervous system neuroinflammatory disorders when clinically indicated. Such testing was not routinely performed in patients with Guillain–Barré syndrome because neuronal antibody evaluation was not considered clinically relevant for peripheral nervous system disorders.

### 2.4. Therapeutic Plasma Exchange

Immunomodulatory treatment strategies were individualized and determined by the treating pediatric neurologists and pediatric intensivists according to disease severity, clinical course, and treatment response. In most patients, first-line therapy consisted of high-dose intravenous methylprednisolone and/or intravenous immunoglobulin (IVIG). Rituximab was administered to selected patients with refractory autoimmune disorders who demonstrated persistent neurological deterioration or an inadequate response despite first-line immunotherapy with high-dose corticosteroids and intravenous immunoglobulin (IVIG), based on multidisciplinary clinical decision-making.

The decision to initiate therapeutic plasma exchange (TPE) was not protocolized and was based on multidisciplinary clinical assessment. In general, TPE was considered in patients with severe neurological involvement, clinical deterioration despite first-line immunotherapy, refractory seizures or status epilepticus, impaired consciousness, progressive neurological deficits, radiological progression, or fulminant disease course.

TPE initiation time was defined as the interval between symptom onset and the first plasma exchange session. All procedures were performed using central venous access and standard institutional apheresis protocols. Approximately 1.0–1.5 estimated plasma volumes were exchanged during each session. Replacement fluid consisted of fresh frozen plasma and/or 5% albumin according to clinical indication, coagulation status, and product availability, with fresh frozen plasma being the predominant replacement fluid in our center. Anticoagulation was performed according to the institutional apheresis protocol, and patients were continuously monitored for hemodynamic stability and procedure-related adverse events throughout each session.

For each patient, the timing of TPE initiation, number of exchange sessions, concomitant immunomodulatory therapies, replacement fluid, anticoagulation strategy, and procedure-related complications were retrospectively recorded.

### 2.5. Classification of TPE Indications

Indications for TPE were categorized according to the 2023 American Society for Apheresis (ASFA) guidelines whenever applicable [6]. Conditions not specifically addressed in the ASFA recommendations were classified according to the Oxford Centre for Evidence-Based Medicine (OCEBM)-based evidence framework described in the British Society for Haematology guideline [10].

### 2.6. Neuroimaging Assessment

Baseline and follow-up magnetic resonance imaging (MRI) studies were reviewed using radiology reports and neuroimaging assessments documented in the medical records. MRI interpretations had originally been performed by pediatric neuroradiologists and/or pediatric neurologists as part of routine clinical care. MRI findings were evaluated for lesion presence, localization, and radiological evolution over time when serial imaging was available. MRI findings were categorized according to the dominant radiological pattern. “Encephalitic changes” were defined as parenchymal abnormalities considered consistent with inflammatory central nervous system involvement, including cortical, subcortical, white matter, deep gray matter, brainstem, cerebellar, or spinal cord lesions. Findings not fulfilling these criteria were classified as non-specific.

For patients with follow-up MRI studies, radiological outcomes were classified as improved (complete or partial resolution of abnormalities), stable, progression, or unavailable when follow-up imaging was not performed.

### 2.7. Outcome Measures

The primary outcome was unfavorable neurological outcome at hospital discharge. Neurological status was assessed using the Pediatric Cerebral Performance Category (PCPC) score. The PCPC score ranges from 1 to 6, with higher scores indicating worse neurological function [11]. Patients with a PCPC score ≥3 and those who died during hospitalization were classified as having an unfavorable neurological outcome. This threshold was selected because a PCPC score of ≥3 represents at least moderate neurological disability and is a widely used, validated, and reproducible neurological outcome measure in pediatric critical care, making it particularly suitable for retrospective cohort studies.

Secondary outcomes included PICU length of stay, requirement for invasive mechanical ventilation, duration of mechanical ventilation, need for vasoactive support, radiological evolution on follow-up MRI, and mortality.

Although more detailed functional assessment tools are available, the PCPC score was selected because of its widespread use in pediatric critical care research, reproducibility, and suitability for retrospective cohort studies.

An exploratory comparison between patients with favorable and unfavorable neurological outcomes was performed.

### 2.8. Statistical Analysis

Statistical analyses were performed using IBM SPSS Statistics version 22.0 (IBM Corp., Armonk, NY, USA). Continuous variables were expressed as median and interquartile range (IQR), whereas categorical variables were presented as frequency and percentage.

Given the descriptive nature of the study and the limited sample size, analyses were primarily exploratory and descriptive.

## 3. Results

### 3.1. Patient Characteristics

A total of 22 pediatric patients who underwent TPE for neuroimmunological disorders during the study period were included. The median age was 104 months (IQR, 62.3–151.8), and 11 patients (50%) were female. The median duration of symptoms before admission was 6 days (IQR, 2.8–9.5). At PICU admission, the median GCS score was 12.5 (IQR, 10–15), while the median PRISM-III and PELOD-2 scores were 0 (IQR, 0–2) and 0 (IQR, 0–1.75), respectively. The median length of PICU stay was 13 days (IQR, 8.5–27.8). Baseline demographic and clinical characteristics are summarized in Table 1.

The most common diagnosis was GBS (n = 6), followed by autoimmune encephalitis (n = 6), infection-triggered encephalopathy syndrome (ITES)/acute necrotizing encephalopathy (ANE) spectrum disorders (n = 4), MOGAD (n = 3), ADEM (n = 2), and MERS (n = 1).

### 3.2. Therapeutic Plasma Exchange Characteristics

The median time from symptom onset to TPE initiation was 9 days (IQR, 6–13), and patients underwent a median of 7 TPE sessions (IQR, 5–10.5). TPE was administered following first-line immunomodulatory therapies, most commonly intravenous methylprednisolone and IVIG. Rituximab was used in selected refractory cases. Individual patient characteristics, treatment details, and outcomes are presented in Table 2.

Nine patients (40.9%) required invasive mechanical ventilation, with ventilation duration ranging from 3 to 77 days. Vasoactive support was required in four patients (18.1%). No major TPE-related complications were observed during the study period.

### 3.3. Cerebrospinal Fluid, Microbiological, and Immunological Findings

CSF analysis was available for patients with central nervous system neuroinflammatory disorders. The median CSF white blood cell count was 4 cells/mm^3^ (IQR, 0–27.5), and the median CSF protein concentration was 0.37 g/L (IQR, 0.24–0.56). Pleocytosis was identified in 8 of 16 patients (50%). Oligoclonal bands were negative in all five patients tested.

Positive CSF PCR findings were identified in two patients. One patient with anti-NMDAR encephalitis had a preceding episode of PCR-confirmed HSV-1 encephalitis and subsequently developed anti-NMDAR encephalitis during a clinical relapse. Another patient diagnosed with ADEM had CSF bocavirus positivity. Both cases were classified according to their overall clinical, radiological, and immunological features rather than CSF PCR findings alone. Serum and/or CSF neuronal antibody testing was performed in 16 patients. Anti-NMDAR antibodies were detected in one patient, anti-GAD antibodies in two patients, and anti-MOG antibodies in three patients. Detailed CSF and autoantibody findings are provided in Table 3 and Supplementary Table S1.

Potential triggering pathogens were identified in four patients with infection-triggered encephalopathy syndromes, including Mycoplasma pneumoniae in two patients, Influenza A in one patient, and SARS-CoV-2 in one patient. In addition, one patient with ADEM had CSF positivity for human bocavirus. Detailed microbiological and immunological findings are presented in Table 3 and Supplementary Table S1.

### 3.4. Neuroimaging Findings

Baseline MRI findings were heterogeneous and included normal examinations, non-specific abnormalities, and encephalitic changes involving cortical, subcortical, deep gray matter, brainstem, cerebellar, and spinal cord regions. Follow-up MRI was available in 13 of 22 patients (59.1%). Among these, complete radiological resolution was observed in 1 patient (7.7%), partial improvement in 3 (23.1%), stability in 6 (46.2%), and progression in 3 (23.1%). Follow-up neuroimaging was not performed in the remaining 9 patients. Detailed MRI evolution is presented in Supplementary Table S2.

### 3.5. Clinical Outcomes

At hospital discharge, favorable neurological outcomes were observed in 14 patients (63.6%), whereas 8 patients (36.4%) had unfavorable neurological outcomes. The median PCPC score at discharge was 2 (IQR, 1–4). Three patients (13.6%) died during hospitalization. Clinical outcomes according to individual diagnoses are summarized in Table 1 and Table 2.

An exploratory comparison between patients with favorable and unfavorable neurological outcomes is presented in Supplementary Table S3. Patients with unfavorable neurological outcomes had significantly lower admission GCS scores and more frequently required invasive mechanical ventilation. Longer time to TPE initiation, higher illness severity scores, and greater vasoactive support requirements were also observed, although these differences did not reach statistical significance.

## 4. Discussion

In this retrospective cohort of critically ill children with neuroimmunological disorders, TPE was utilized across a broad spectrum of inflammatory neurological diseases, including autoimmune encephalitis, demyelinating disorders, GBS, and infection-triggered encephalopathy syndromes. The cohort reflects routine clinical practice in a tertiary pediatric intensive care unit, where TPE was generally reserved for patients with severe neurological involvement, rapidly progressive disease, or insufficient response to first-line immunomodulatory therapies. The broad range in TPE initiation timing (2–61 days after symptom onset) reflects the heterogeneity of underlying diagnoses, referral patterns, treatment strategies, and disease course. Patients with Guillain–Barré syndrome generally underwent TPE after an inadequate response to IVIG, whereas those with autoimmune encephalitis often received corticosteroids and IVIG before escalation to plasma exchange. In contrast, fulminant encephalopathy syndromes typically prompted earlier TPE because of rapid neurological deterioration.

The available evidence supporting TPE in pediatric neuroimmunological disorders remains limited. Previous studies have predominantly consisted of retrospective single-center series or disease-specific cohorts. Eyre et al. provided one of the most comprehensive reviews to date, summarizing indications, levels of evidence, and safety across a broad spectrum of pediatric neurological disorders [5]. More recently, Eichinger et al. reported the complication profile and clinical outcomes of TPE in a single-center pediatric neuroimmune cohort, demonstrating a high rate of clinical improvement despite predominantly mild-to-moderate procedure-related complications [7]. Collectively, these studies underscore the limited pediatric-specific evidence base and highlight that current clinical practice is frequently extrapolated from adult data or guided by expert consensus. Our cohort extends the existing literature by describing real-world TPE utilization across a particularly heterogeneous population of critically ill children, including several disorders that are underrepresented in previous pediatric TPE series.

One of the principal observations of this study is the marked heterogeneity of both diagnoses and levels of evidence supporting TPE use. While TPE is well established for disorders such as GBS and autoimmune encephalitis, evidence remains substantially more limited for severe encephalopathy syndromes, including ANE and infection-triggered encephalopathy syndromes (ITES). Approximately one-quarter of our cohort consisted of conditions not specifically addressed in current ASFA recommendations [6]. This finding highlights a common challenge in pediatric neurocritical care, where treatment decisions frequently rely on pathophysiological rationale, disease severity, and multidisciplinary clinical judgment rather than high-quality disease-specific evidence.

This issue was particularly relevant for patients with ANE- and AESD-like/ITES presentations. Although these disorders are not specifically included in current ASFA recommendations, accumulating evidence suggests that severe cases involve cytokine dysregulation, blood–brain barrier disruption, and secondary neuroinflammation [12,13,14]. Consequently, TPE may be considered as a rescue strategy aimed at removing circulating inflammatory mediators and modulating systemic immune activation. However, the evidence supporting this approach remains limited and is largely derived from case reports, small case series, and expert opinion. The inclusion of these patients in our cohort illustrates the gap between real-world clinical practice and the currently available evidence base for many severe pediatric encephalopathy syndromes. Radiological evolution did not consistently parallel neurological outcome in our cohort. Notably, radiological progression was not invariably associated with an unfavorable clinical outcome, suggesting that MRI findings should be interpreted in conjunction with the overall clinical course rather than as an isolated indicator of treatment response.

Consistent with previous reports, potential infectious triggers identified in our cohort included Influenza A, *Mycoplasma pneumoniae*, and SARS-CoV-2. Influenza infection has been most strongly associated with ANE, whereas Mycoplasma pneumoniae has been linked to a broader spectrum of infection-triggered encephalopathy syndromes, including AESD-like and ANE-like presentations [2,15]. These observations further support the concept that diverse infectious pathogens may trigger convergent neuroinflammatory pathways leading to severe encephalopathic syndromes.

At hospital discharge, favorable neurological outcomes were observed in 63.6% of patients, whereas 36.4% had unfavorable neurological outcomes, defined as a PCPC score of ≥3 or death. Mortality occurred in 13.6% of patients. These findings should be interpreted within the context of a highly selected cohort of critically ill children rather than as a measure of TPE efficacy. Consistent with this, Eyre et al. noted that while TPE may offer clinical stabilization in acute neuroimmunological disorders, outcomes vary considerably according to disease severity and treatment responsiveness, with refractory cases carrying a less favorable prognosis [5]. Similarly, Eichinger et al. reported clinical improvement in 84% of pediatric patients undergoing TPE for neuroimmunological disorders [7]. The more favorable outcomes observed in that cohort may partly reflect differences in case mix, as the study population was predominantly composed of relapsing demyelinating disorders, whereas severe encephalopathic syndromes represented a larger proportion of patients in our cohort.

GBS represented the largest diagnostic subgroup in our cohort and was the only indication supported by the highest level of evidence for TPE use (ASFA Category I, Grade 1A). All patients received IVIG before TPE initiation, reflecting the use of plasma exchange as an escalation strategy in the setting of persistent or progressive neurological deficits. No deaths occurred in this subgroup, and neurological outcomes were generally favorable. These findings are consistent with previous pediatric GBS studies reporting good functional recovery despite residual deficits in a subset of patients [16,17]. Although randomized pediatric data have demonstrated shorter durations of mechanical ventilation with TPE compared with IVIG in mechanically ventilated children, short-term functional outcomes appear broadly similar between treatment modalities [18]. Given the descriptive nature of our study and the absence of a comparator group, no conclusions regarding the comparative effectiveness of TPE can be drawn.

Outcomes among patients with autoimmune encephalitis were variable, with most patients experiencing unfavorable neurological outcomes at hospital discharge. However, recovery frequently extends beyond the acute hospitalization period, and substantial neurological improvement may occur during long-term follow-up [19,20]. Accordingly, discharge PCPC scores should be interpreted as reflecting neurological status at a single time point rather than definitive long-term prognosis. This consideration also applies to other immune-mediated demyelinating disorders, including MOGAD and ADEM, in which neurological recovery may continue for months after hospital discharge. Previous studies have also suggested that early plasma exchange may be associated with improved outcomes in pediatric autoimmune encephalitis [21].

The interpretation of positive CSF PCR findings in neuroinflammatory disorders may also be challenging [22,23]. One patient with anti-NMDAR encephalitis had a preceding episode of PCR-confirmed HSV-1 encephalitis and subsequently developed clinical and immunological features consistent with secondary autoimmune encephalitis during relapse, in keeping with the recognized phenomenon of post-herpetic autoimmune encephalitis [24]. Another patient classified as ADEM had CSF bocavirus positivity; however, the combination of encephalopathy, multifocal MRI abnormalities involving both white matter and deep gray matter structures, limited CSF inflammation, and complete neurological recovery was considered more consistent with an immune-mediated demyelinating process than with primary viral encephalitis [25]. These cases underscore the importance of integrating microbiological, clinical, radiological, and immunological findings when establishing diagnostic classifications in pediatric neuroinflammatory disorders. Mortality was confined to patients with severe encephalopathic syndromes, highlighting the poor outcomes that may accompany fulminant pediatric encephalopathy.

## 5. Limitations

This study has several limitations. The retrospective single-center design introduces the possibility of selection bias and limits generalizability. The study population was heterogeneous and included disorders with distinct pathophysiological mechanisms, disease trajectories, and levels of evidence supporting TPE use. Neuroimaging findings were derived from retrospective clinical assessments and were not subject to blinded centralized review. The decision to initiate TPE was not protocolized and was determined individually by treating clinicians, introducing potential indication bias. Furthermore, all patients received concomitant immunomodulatory therapies, precluding assessment of the independent therapeutic contribution of plasma exchange. Neurological outcomes were assessed at hospital discharge, and long-term functional outcomes were not systematically available. Finally, the absence of a control group precludes causal inference regarding the effectiveness of TPE; therefore, our findings should be interpreted primarily as reflecting real-world clinical practice rather than demonstrating treatment efficacy.

## 6. Conclusions

Despite these limitations, our study provides a detailed description of real-world TPE utilization in a diverse cohort of critically ill pediatric patients with neuroimmunological disorders. Given the rarity of many of these conditions, particularly infection-triggered encephalopathy syndromes and other disorders for which evidence remains limited, pediatric data are scarce. Future multicenter collaborative studies with standardized treatment protocols and longitudinal outcome assessment are needed to better define the role, timing, and patient selection criteria for TPE in pediatric neuroimmunology.

## Figures and Tables

**Table 1:** Baseline demographic and clinical characteristics of patients undergoing TPE (n = 22).

Characteristics	n (% or Median [IQR])
Female	11 (50)
Age (months)	104 (62.25–151.75)
Symptom duration before admission (days)	6 (2.75–9.5)
Admission Glasgow Coma Scale (GCS)	12.5 (10–15)
PRISM-III score	0 (0–2)
PELOD-2 score	0 (0–1.75)
PICU length of stay, days	13 (8.5–27.75)
**Diagnosis**	
GBS	6 (27.3)
Autoimmune encephalitis	6 (27.3)
ITES/ANE spectrum	4 (18.2)
MOGAD	3 (13.6)
ADEM	2 (9.1)
MERS	1 (4.5)
**TPE procedures**	
Median number of TPE sessions per patient	7 (5–10.5)
Time to TPE initiation (days)	9 (6–13)
**Organ support**	
Mechanical ventilation requirement	9 (40.9)
Vasoactive support	4 (18.1)
**Clinical outcomes**	
Unfavorable neurological outcome, n (%)	8 (36.4)
Favorable neurological outcome, n (%)	14 (63.6)
Mortality, n (%)	3 (13.6)
PCPC score at discharge	2 (1–4)

TPE initiation time was defined as the number of days from symptom onset to the first plasma exchange session. Continuous variables are presented as median (interquartile range). Categorical variables are presented as number (percentage). Outcome was classified according to discharge PCPC score. Unfavorable neurological outcome was defined as PCPC ≥ 3 or death. **Abbreviations:** TPE, therapeutic plasma exchange; PCPC, Pediatric Cerebral Performance Category; PRISM-III, Pediatric Risk of Mortality III; PELOD-2, Pediatric Logistic Organ Dysfunction 2; GCS, Glasgow Coma Scale; PICU, pediatric intensive care unit.

**Table 2:** Individual patient characteristics, TPE details, and outcomes.

Patient Number	Age ^†^	Sex	Diagnosis	IMV ^§^	Treatments	Evidence Level of Indication ^¶^	TPE Initiation Days *	Number of TPE Procedures	Baseline MRI	MRI Evolution	Length of Stay in PICU ^§^	Outcome ^#^
1	160	M	Seronegative AE	-	MPS-IVIG-TPE	I-1C	61	5	Normal	UA	5	No sequelae
2	105	F	ADEM	5	MPS-IVIG-TPE	II-2C	11	13	Encephalitic	Partial improvement	27	Sequelae
3	73	F	Anti-GAD AE	-	MPS-IVIG-TPE- RTX	I-1C	8	12	Non-specific	Stable	27	Sequelae
4	72	M	Anti-GAD AE	3	MPS-IVIG-TPE- RTX	I-1C	28	15	Non-specific	Stable	16	Sequelae
5	149	F	Probable AESD-like ITES	77	MPS-IVIG-TPE- RTX	OCEBM-4	10	15	Normal	Progression	87	Death
6	168	M	Seronegative AE	4	MPS-IVIG-TPE	I-1C	2	7	Encephalitic	UA	12	No sequelae
7	16	M	ANE	-	MPS-IVIG-TPE	OCEBM-4	4	5	Encephalitic	Partial improvement	12	Sequelae
8	25	F	Anti-NMDAR encephalitis	6	MPS-IVIG-TPE	I-1B	26	10	Encephalitic	Progression	41	Death
9	86	F	ANE-like ITES	52	MPS-IVIG-TPE	OCEBM-4	16	10	Encephalitic	Stable	52	Sequelae
10	192	F	MOGAD	-	MPS-IVIG-TPE- RTX	II-2C	8	7	Encephalitic	Progression	30	No sequelae
11	52	F	ADEM	-	IVIG-MPS-TPE	II-2C	41	7	Encephalitic	Partial improvement	6	No sequelae
12	121	F	Seronegative AE	40	MPS-IVIG-TPE	I-1C	6	9	Encephalitic	Stable	57	No sequelae
13	51	M	MOGAD	-	MPS-IVIG-TPE	II-2C	3	10	Encephalitic + spinal cord	Complete resolution	12	No sequelae
14	122	M	Unclassifiable ITES with ANE-like feature	17	MPS-IVIG-TPE	OCEBM-4	12	1	Encephalitic	Stable	18	Death
15	168	M	MERS	-	IVIG-MPS-TPE	OCEBM-4	8	5	Encephalitic	Stable	14	No sequelae
16	116	M	MOGAD	-	IVIG-MPS-TPE	II-2C	6	5	Normal	UA	9	No sequelae
17	54	F	GBS	-	IVIG-TPE	I-1A	9	12	Normal	UA	14	No sequelae
18	103	F	GBS	-	IVIG-TPE	I-1A	9	5	Normal	UA	9	No sequelae
19	98	M	GBS	4	IVIG-TPE	I-1A	9	7	Normal	UA	7	No sequelae
20	148	F	GBS	-	IVIG-TPE	I-1A	10	5	Normal	UA	5	No sequelae
21	65	M	GBS	-	IVIG-TPE	I-1A	9	5	Normal	UA	6	No sequelae
22	184	M	GBS	-	IVIG-TPE	I-1A	3	10	Normal	UA	10	No sequelae

* TPE initiation time was defined as the number of days from symptom onset to the first plasma exchange session. ^†^ Age is presented in months. ^§^ Duration of invasive mechanical ventilation and length of PICU stay are presented in days. ^¶^ I-1A, I-1B, I-1C, and II-2C indicate the ASFA category and recommendation grade; OCEBM-4 indicates Oxford Centre for Evidence-Based Medicine Level 4 evidence. ^#^ Patients with a PCPC score ≥ 3 and patients who died during hospitalization were classified as having an unfavorable neurological outcome. Abbreviations: AE, autoimmune encephalitis; ADEM, acute disseminated encephalomyelitis; ANE, acute necrotizing encephalopathy; GBS, Guillain–Barré syndrome; IMV, invasive mechanical ventilation; IVIG, intravenous immunoglobulin; MERS, mild encephalitis/encephalopathy with a reversible splenial lesion; MOGAD, myelin oligodendrocyte glycoprotein-associated disease; MPS, methylprednisolone; PICU, pediatric intensive care unit; RTX, rituximab; TPE, therapeutic plasma exchange.

**Table 3:** Cerebrospinal Fluid and Neuronal Autoantibody Findings.

Variable	Value
**CSF findings**	
CSF white blood cell count, cells/mm^3^	4 (0–27.5)
Pleocytosis, n (%)	8 (50)
CSF protein, g/L	0.37 (0.24–0.56)
CSF glucose, mg/dL	71.8 (60.1–83.0)
Oligoclonals bands positive, n/N	0/5
Positive CSF microbiological finding, n (%)	2 (17.6)
Identified CSF pathogens	HSV-1 (n = 1), Bocavirus (n = 1)
**Neuronal antibody testing**	
Serum and/or CSF neuronal antibody testing performed, n (%)	16/16 (100)
CSF anti-NMDAR positive, n (%)	1 (5.9)
CSF anti-GAD positive, n (%)	2 (11.8)
CSF anti-MOG positive, n (%)	3 (17.6)

Data are presented as median (IQR) or n (%), unless otherwise specified. CSF: cerebrospinal fluid; NMDAR: N-methyl-D-aspartate receptor; GAD: glutamic acid decarboxylase; MOG: myelin oligodendrocyte glycoprotein. n/N indicates the number of positive patients among those tested. Among patients with CNS neuroinflammatory disorders, serum and/or CSF neuronal antibody testing was performed in all 16 patients (100%).

## Data Availability

The datasets generated and/or analyzed during the current study are available from the corresponding author on reasonable request.

## Supplementary Materials

The following supporting information can be downloaded at https://www.mdpi.com/article/10.3390/children13070908/s1, Table S1: Cerebrospinal Fluid and Autoantibody Characteristics of Patients with Central Nervous System Neuroinflammatory Disorders; Table S2: Evolution of MRI Findings Following Therapeutic Plasma Exchange; Table S3: Exploratory comparison of baseline clinical characteristics according to neurological outcome at hospital discharge.

## Abbreviations

ADEM	Acute disseminated encephalomyelitis
AE	Autoimmune encephalitis
AESD	Acute encephalopathy with biphasic seizures and late reduced diffusion
ANE	Acute necrotizing encephalopathy
ASFA	American Society for Apheresis
CNS	Central nervous system
CSF	Cerebrospinal fluid
GBS	Guillain–Barré syndrome
GCS	Glasgow Coma Scale
HIS	Health Information System
HSV-1	Herpes simplex virus type 1
IMV	Invasive mechanical ventilation
IQR	Interquartile range
ITES	Infection-triggered encephalopathy syndrome
IVIG	Intravenous immunoglobulin
MERS	Mild encephalitis/encephalopathy with a reversible splenial lesion
MOGAD	Myelin oligodendrocyte glycoprotein antibody-associated disease
MRI	Magnetic resonance imaging
NMDAR	N-methyl-D-aspartate receptor
OCB	Oligoclonal bands
OCEBM	Oxford Centre for Evidence-Based Medicine
PCR	Polymerase chain reaction
PCPC	Pediatric Cerebral Performance Category
PELOD-2	Pediatric Logistic Organ Dysfunction-2
PICU	Pediatric intensive care unit
PRISM III	Pediatric Risk of Mortality III
RTX	Rituximab
SARS-CoV-2	Severe acute respiratory syndrome coronavirus 2
TPE	Therapeutic plasma exchange
WBC	White blood cell
