# Supplementary material for: Therapeutic Plasma Exchange in Critically Ill Children with Neuroimmunological Disorders: A Single-Center Cohort Study
Source: Children (Basel). 2026 Jul 9;13(7):908. doi: 10.3390/children13070908 (PMC13407348; doi:10.3390/children13070908)
Supplement: Supplementary file 1 [file children-13-00908-s001.zip › children-4402418-supplementary.pdf]

Supplementary Table S1. Cerebrospinal Fluid and Autoantibody Characteristics of Patients with Central Nervous System Neuroinflammatory Disorders.

Patient	Diagnosis	CSF WBC (/mm³)	CSF Protein (g/L)	OCB	CSF PCR	Probable Triggering Pathogen	Autoantibody
1	Seronegative AE	0	0.42	Negative	Negative		Negative
2	ADEM	21	0.743	Negative	Negative		Negative
3	Anti-GAD AE	0	0.31	Not tested	Negative		Anti-GAD +
4	Anti-GAD AE	1	0.256	Negative	Negative		Anti-GAD +
5	Probable AESD-like ITES	2	0.154	Not tested	Negative	*M. pneumonia*	Negative
6	Seronegative AE	0	0.47	Not tested	Negative		Negative
7	ANE	0	0.515	Not tested	Negative	Influenza A	Negative
8	Anti-NMDAR encephalitis	0	0.120	Not tested	HSV-1		Anti-NMDAR +
9	ANE-like ITES	60	0.688	Not tested	Negative	*M. pneumonia*	Negative
10	MOGAD	47	0.413	Not tested	Negative		Anti-MOG +
11	ADEM	8	0.35	Not tested	Human bocavirus		Negative
12	Seronegative AE	14	0.184	Not tested	Negative		Negative
13	MOGAD	68	0.316	Not tested	Negative		Anti-MOG +
14	Unclassifiable ITES with ANE-like features	11	2.02	Negative	Negative		Negative
15	MERS	0	0.37	Not tested	Negative	SARS-CoV-2	Negative
16	MOGAD	6	0.38	Negative	Negative		Anti-MOG +

Abbreviations: *AE, autoimmune encephalitis; ADEM, acute disseminated encephalomyelitis; ANE, acute necrotizing encephalopathy; AESD, acute encephalopathy with biphasic seizures and late reduced diffusion; ITES, infection-triggered encephalopathy syndrome; MERS, mild encephalitis/encephalopathy with a reversible splenial lesion; MOGAD, myelin oligodendrocyte glycoprotein antibody-associated disease; CSF, cerebrospinal fluid; WBC, white blood cell; OCB, oligoclonal bands; PCR, polymerase chain reaction; HSV-1, herpes simplex virus type 1; SARS-CoV-2, severe acute respiratory syndrome coronavirus 2; Anti-GAD, glutamic acid decarboxylase antibody; Anti-NMDAR, N-methyl-D-aspartate receptor antibody; Anti-MOG, myelin oligodendrocyte glycoprotein antibody.*

Supplementary Table S2. Evolution of MRI Findings Following Therapeutic Plasma Exchange.

MRI outcome	n (%)
Complete resolution	1 (7.7)
Partial improvement	3 (23.1)
Stable	6 (46.2)
Progression	3 (23.1)
Unavailable	9 (-)

MRI follow-up was available in 13 of 22 patients (59.1%). Percentages are calculated among patients with available follow-up imaging. Follow-up neuroimaging was not performed in the remaining 9 patients.

Supplementary Table S3. Exploratory comparison of baseline clinical characteristics according to neurological outcome at hospital discharge.

	Neurological outcome		**p value**
	Favorable neurological outcome (n=14)	Unfavorable neurological outcome (n=8)	
Symptom duration before admission, days	4.5 (2.0-7.25)	7 (4-22.25)	0.212
Admission GCS	14.5 (10.75-15)	10 (8.25-12.75)	**0.016**
PRISM-III score	0 (0-0)	2.5 (0-5)	0.127
PELOD-2 score	0 (0-0)	1 (0-4)	0.212
Invasive mechanical ventilation, n (%)	3 (21.4)	6 (75.0)	**0.014**
Vasoactive support, n (%)	1 (7.1)	3 (37.5)	0.076
Time to TPE initiation, days	8 (5.25-9.25)	11.5 (8.5-23.5)	0.095

Data are presented as median (interquartile range) or n (%), as appropriate. Favorable neurological outcome was defined as a discharge Pediatric Cerebral Performance Category (PCPC) score <3, whereas unfavorable neurological outcome was defined as a discharge PCPC score ≥3 or death. Continuous variables were compared using the Mann–Whitney U test and categorical variables using Fisher's exact test. Owing to the small sample size, these analyses should be considered exploratory.
